# Refining manual annotation effort of acoustic data to estimate bird species richness and composition: The role of duration, intensity, and time

**DOI:** 10.1002/ece3.9491

**Published:** 2022-11-14

**Authors:** Taylor Shaw, Sina‐Rebekka Schönamsgruber, João M. Cordeiro Pereira, Grzegorz Mikusiński

**Affiliations:** ^1^ Geobotany, Faculty of Biology University of Freiburg Freiburg Germany; ^2^ Chair of Wildlife Ecology and Management University of Freiburg Freiburg Germany; ^3^ School for Forest Management Swedish University of Agricultural Sciences Skinnskatteberg Sweden

**Keywords:** acoustic survey, annotation, bioacoustic, bird richness and composition, forest birds, passive acoustic monitoring, point count, survey design

## Abstract

Manually annotating audio files for bird species richness estimation or machine learning validation is a time‐intensive task. A premium is placed on the subselection of files that will maximize the efficiency of unique additional species identified, to be used for future analyses. Using acoustic data collected in 17 plots, we created 60 subsetting scenarios across three gradients: intensity (minutes in an hour), day phase (dawn, morning, or both), and duration (number of days) for manual annotation. We analyzed the effect of these variables on observed bird species richness and assemblage composition at both the local and entire study area scale. For reference, results were also compared to richness and composition estimated by the traditional point count method. Intensity, day phase, and duration all affected observed richness in decreasing respective order. These variables also significantly affected observed assemblage composition (in the same order of effect size), but only the day phase produced compositional dissimilarity that was due to phenological traits of individual bird species, rather than differences in species richness. All annotation scenarios requiring equal sampling effort to point counts yielded higher species richness than the point count method. Our results show that a great majority of species can be obtained by annotating files at high sampling intensities (every 3 or 6 min) in the morning period (post‐dawn) over a duration of two days. Depending on a study's aim, different subsetting parameters will produce different assemblage compositions, potentially omitting rare or crepuscular species, species representing additional functional groups and natural history guilds, or species of higher conservation concern. We do not recommend one particular subsetting regime for all research objectives, but rather present multiple scenarios for researchers to understand how intensity, day phase, and duration interact to identify the best subsetting regime for one's particular research interests.

## Graphical abstract

This study examines the effect of 60 subsampling scenarios on the resulting observed richness and composition estimates of bird assemblages. Results demonstrate how time of morning, duration, and recording intensity differentially affect resulting metrics. Results also identify the most efficient scenarios for accumulating species richness, all of which outperform the traditional point count method with equal or less time effort.

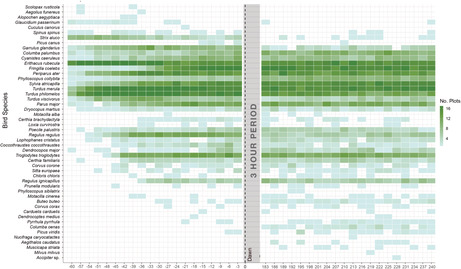

## 1 INTRODUCTION

The traditional method for monitoring birds in forest habitats is the point count method, whereby a human observer travels into the field and observes all birds seen or heard within a particular distance radius for a set amount of time (usually 5–20 min; Green et al., 2010; Ralph et al., 1995). An increasingly popular alternative to point count surveys is to observe vocalizing birds through passive acoustic monitoring (PAM), which entails continuous or semi‐continuous recording at a site, after which time expert observers listen to acoustic files, often with the visual aid of spectrograms, in order to determine species identities. This method can be used for the estimation of assemblage richness and composition, and has vastly grown in popularity due to the falling costs of autonomous recording units, increasing ease of data collection and storage, and extensive evidence supporting its comparability, and even superiority, to traditional survey methods (Darras et al., 2018). PAM is particularly attractive in forest environments where almost all detections during point counts are done by auditory cues (Brewster & Simons, 2009).

Advances in machine learning algorithms have been able to leverage the increasing ease of PAM and employ it on larger spatial and temporal scales than manual file annotation alone can achieve, as the processing of large volumes of acoustic data is becoming increasingly efficient (Joppa, 2017; Stowell et al., 2019). Limitations to automatic classification include generalizability to unmatched conditions, the availability of large previously annotated datasets, low accuracy, low robustness to noise such as wind and rain, the need for manual tuning of algorithm parameters, post‐processing of results, and sufficient expertise in machine learning (Stowell et al., 2019). However, increasingly sophisticated detection algorithms have demonstrated their ability to overcome many of these obstacles (Kahl et al., 2021; Stowell et al., 2019; Wood et al., 2021), and progress in this field is rapidly advancing (Denton et al., 2022; Huang & Basanta, 2021; Liu et al., 2022), particularly as more manually annotated files are added to existing datasets (Wood et al., 2022; Zhong et al., 2021) that researchers can use without building new algorithms. This method is particularly promising because once a classifier can produce robust results for a particular area, automated (and theoretically continuous) monitoring becomes possible, overcoming the current considerable limitation of time available for manual annotation alone to estimate which species occupy that area over time.

Until machine learning algorithms are improved for ubiquitous use for automated bird identification, PAM research is occurring in a transition period whereby bird monitoring programs use point counts, bioacoustic identification, machine learning classifiers, or some combination thereof. Due to the substantial learning curve for using classifiers (Stowell et al., 2019), or the desire to maintain comparable long‐term datasets derived from point counts (Brlík et al., 2021; Sauer et al., 2013), machine learning classifiers are not ubiquitously adopted. In other cases, there is a dearth of annotated data from certain regions (de Araújo, 2013; xeno‐canto, 2022), which is required to build accurate classifiers; in such cases, manually annotated data will be a necessary precursor to scalable machine learning campaigns in those areas. For these reasons, manual annotation is and will continue to be an important link as our knowledge is slowly transitioning to digital and automated monitoring methods (Symes et al., 2022). Manual annotations are, however, time‐consuming (Rempel et al., 2005; Swiston & Mennill, 2009; Wimmer et al., 2013), and researchers must maximize this time by subsetting acoustic data efficiently to identify the maximum number of unique species without repeatedly identifying species already observed in that area. Whether manual annotations from PAM are used to directly estimate species richness or to validate the predictions of machine learning algorithms (reducing false positives), it is recommended that this time be strategically allocated to maximize accuracy for either objective (Symes et al., 2022).

Several studies have recently compared different recording schedules, or different subsetting scenarios from continuous audio, to establish optimal parameters for estimating bird species richness. The consensus is that increasing the number of days, as well as the number of h per day, increases species richness estimates (de Araújo et al., 2021; Sugai et al., 2020; Wimmer et al., 2013; Wood et al., 2021). Despite this generalized trend that more time investment in manual annotation yields higher species richness, specific recommendations for recording schedules or subsetting from continuous audio vary by region due to different bird communities and their respective probabilities of detection (Cook & Hartley, 2018; Drake et al., 2021; La & Nudds, 2016; Wood et al., 2021). There is an increasing need, therefore, for regionally specific sampling curves for researchers to make evidence‐based decisions about how much sampling time is sufficient for their research goals, given the resources at hand. To date, no study has produced these curves for central European forests, where there is an increasing interest in evaluating how forest management affects forest bird assemblages (Basile, Asbeck, et al., 2021; Basile, Storch, et al., 2021; Storch et al., 2020; Thorn et al., 2020). Birds are extensively used as environmental and biodiversity indicators (Devictor et al., 2008; Gregory et al., 2005; Stephens et al., 2016). As forest birds have suffered continent‐wide decline over the last 40 years (Burns et al., 2021), there is an ongoing effort to transition toward more biodiversity‐friendly silvicultural approaches (e.g., Gustafsson et al., 2020; Vítková et al., 2018), which have locally proved helpful to reverse those declines (Knaus et al., 2018); efficient, scalable monitoring methods such as PAM, whether by bioacoustic identification or annotation for machine learning algorithms, can enable the evaluation of their effectiveness.

Annotated audio files can also yield compositional data, and addressing differences in species composition between different locations, including beta diversity estimates such as species nestedness and turnover, are important questions in contemporary conservation biology (Socolar et al., 2016). In the case of bird assemblages, PAM has the potential to be a wellspring of information for assessing the impact of human activities on assemblage homogenization or heterogenization (also concerning functional diversity) and thereby guide bird conservation decision‐making (Gasc et al., 2017). To our knowledge, no studies exist that also directly address how perceived bird species composition is affected by acoustic file selection choices, but such investigations are recommended (Symes et al., 2022). Wood et al. (2021) addressed species composition insofar as they used simulated bird assemblages to investigate how assemblage structure affects richness estimates depending on how many theoretically rare species comprise the assemblage. However, this study did not address the potential species‐specific differences in assemblage compositions yielded by differing design parameters. Numeric richness can remain unchanged between recording scenarios, while the species identities between them vary (i.e., species turnover). Conversely, species richness can vary between sites, although the composition does not significantly vary, as all the species in the species‐poorer site are also found in the species‐richer site (i.e., nestedness). Shaw, Hedes, et al. (2021) found evidence of compositional differences between bird assemblages (turnover) even when their richness estimates did not differ. However, this difference was found between point counts and bioacoustic methods and not different acoustic monitoring scenarios, so the effect of annotation effort allocation on resulting species composition remains unknown.

In temperate European bird communities, among other regions, the daily onset of bird vocalizations in spring is predictable due in large part to light (Gil & Llusia, 2020) and meteorological conditions (Bruni et al., 2014; Leopold & Eynon, 1961). In good weather, onset of (mostly) male vocalizations culminates in a crescendo of birdsong, a phenomenon known as the dawn chorus, after which birds continue to call throughout the morning, but less intensely. It is also well‐established that the time of vocalization onset varies strongly across species (Leopold & Eynon, 1961; Thomas et al., 2002). We therefore expect the probability of detecting a given species to vary with the day phase depending on multiple interacting factors. Testing the effects of annotation effort allocation solely on bird richness will not adequately capture the species‐specific variation in detection probability.

The aim of our study, therefore, is to analyze richness and compositional differences between acoustic subsetting scenarios across three choice gradients, revealing the potentially missed detection opportunities and across biologically relevant time periods. We not only replicate previous research for a central European forest context for how effort allocation scenarios (hereafter scenarios) affect species richness (Cook & Hartley, 2018; de Araújo et al., 2021; La & Nudds, 2016; Symes et al., 2022; Wimmer et al., 2013; Wood et al., 2021) but we also additionally analyze how these scenarios affect revealed assemblage composition. To do this we considered three main parameters of acoustic file selection: (1) recording intensity, i.e., number of minutes within 1 h; (2) day phase, i.e., period of the morning in relation to sunrise (dawn, morning, or both); and (3) duration, i.e., number of days included in the subset. Lastly, to provide a through line to the most traditional bird monitoring method, we compare our richness and compositional data to the same data yielded from point count surveys.

## 2 MATERIALS AND METHODS

### 2.1 Study area

Our study area is located in the Black Forest (Southwest Germany) and contains 135 1‐ha forest plots, selected within the framework of the Research Training Group ConFoBi (Storch et al., 2020). The plots are distributed across a mosaic landscape with forests dominated by Norway spruce (*Picea abies*), European beech (*Fagus sylvatica*), and silver fir (*Abies alba*). Twenty‐six plots were selected for this study according to their uniformity of shrub layer cover, standing dead wood, and age class, while selecting a range of proportions of broadleaved and coniferous tree species that are representative of the region (see Appendix 1 for more details). Fieldwork took place during peak bird breeding season between April 26, 2021, and June 20, 2021, after foliation had occurred. The plots have mostly closed canopies and are comprised of mature stands >65 years with a history of uneven‐aged silvicultural management, thus they represent a typical Central European temperate mixed montane forest. Altitudes varied from 504 to 1069 m.a.s.l. and the minimum distance between plots was 750 m.

### 2.2 Acoustic recordings

The devices used for this study were Bioacoustic Audio Recorders (BARs) (Frontier Labs Australia), equipped with an omnidirectional microphone (frequency response of ±2 dB from 80 Hz to 20 kHz) and 3.6 V rechargeable batteries supplied by the same company. Audio files were recorded at 22.05 kHz sample rate and saved as 16‐bit WAV format on SanDisk 32 GB SD cards. The dB gain was set to 40 (see Appendix 1 for details on gain settings and handling irregularities due to installation error).

We rotated the BARs through the plots according to their elevation and aspect (from lowest to highest elevation and southerly to northerly aspect) so that each recorder was placed for a week in a given plot. The following week those plots were visited in the same order, one per day, for retrieval, at which point the SD card was removed and batteries recharged for use in another plot. Each BAR was attached 1.5 m high on the center‐most beech tree in each plot, with the microphone oriented perpendicular to the slope, if a slope was detectable (Shaw, Hedes, et al., 2021). They were programmed to record continuously from 04:30 a.m. to 11:00 a.m. to capture bird vocalizations as early as 1 h before sunrise to 4 h after sunrise, accounting for shifts in the sunrise.

### 2.3 Acoustic data processing

**FIGURE 1:**
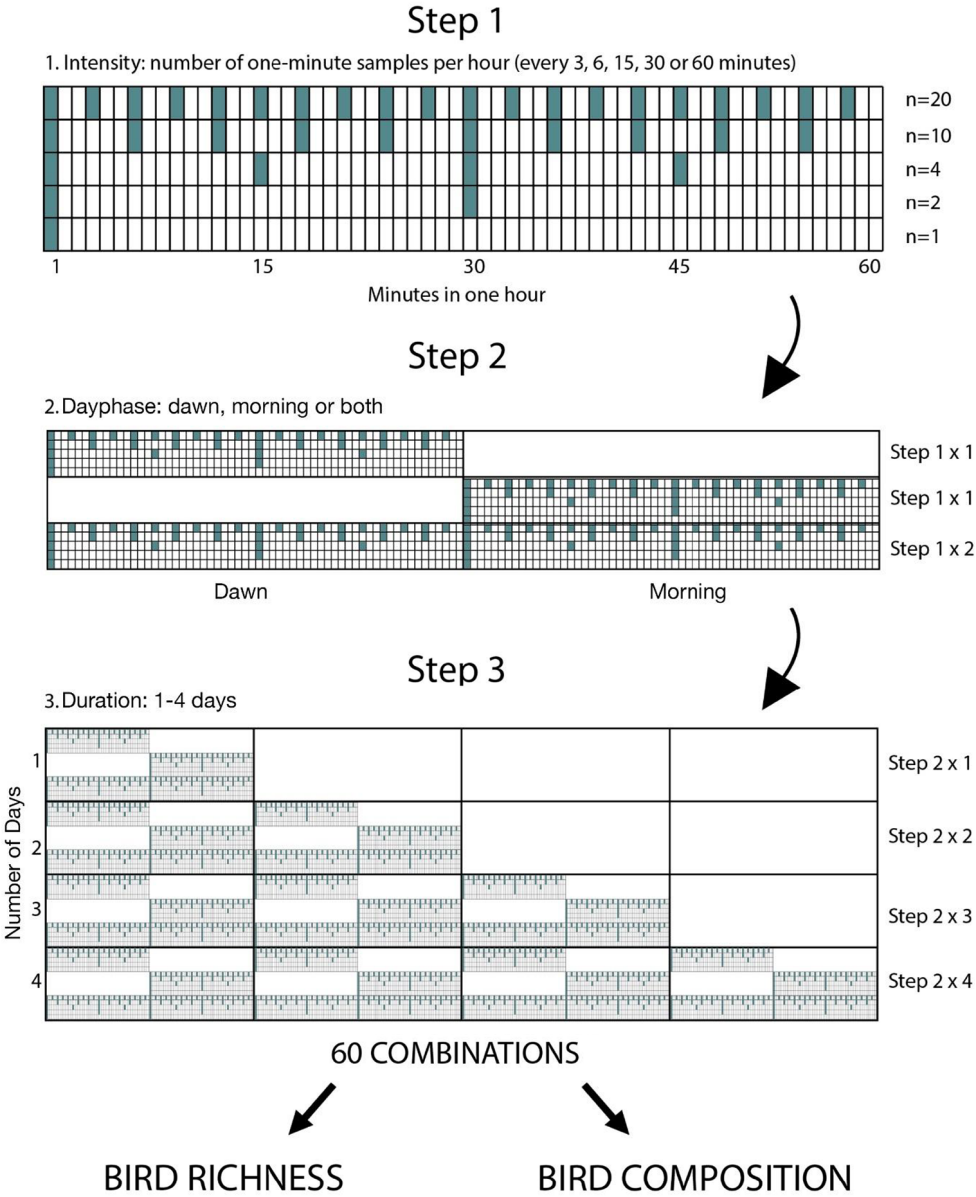
A conceptual framework for the selection of the nested acoustic recording scenarios included in this study. Each row is one scenario, cumulating in 60 different intensity–day phase–duration combinations, which result in a gradient of 1–160 one‐minute files used for manual annotation.

The gradients of independent variables in this study were intensity, day phase, and duration. Machine learning algorithms can automatically process continuous audio; only a subset is typically used for validation or bioacoustic identification purposes. Therefore, to determine our first gradient, intensity, we chose differing subsets of discontinuous audio from our continuous dataset. Discontinuous recordings are subject to less variability caused by clustering of conspecific calls and flocking, and have been shown to be more efficient in detecting the same number of species (Cook & Hartley, 2018), increasing the rate at which the total number of species accumulates. For discontinuous audio subsets, the length of audio is a consideration. Past studies ranged from 10 s (Cook & Hartley, 2018) to 10 min (La & Nudds, 2016), so we standardized this variable by using 1‐min samples. Consequently, we defined our intensity gradient at discontinuous intervals of 1‐min recordings “every 3 min” (*n* = 20 per hour) at the highest intensity, and “every 60 min” (*n* = 1 per hour) at the lowest (Step 1, Figure 1).

Our day phase variable defines two distinct periods: the dawn period is the hour before sunrise, and the morning period is the 1‐h period beginning 3 h after sunrise. Bird vocalizations begin before sunrise during civil and nautical twilight (Bruni et al., 2014), and birds are traditionally surveyed (via point counts) in the morning. To maximize the potential difference between dawn and morning, we elected for non‐consecutive time periods, or 1 h at either end of the wider bird monitoring period. The resulting variable includes dawn alone, morning alone, or the entire combined period including both day phases (Step 2, Figure 1).

To create our duration gradient, we removed the first and last day of the recordings due to any potential effects of the researcher presence in the plot. Given that rain events were commonplace in spring, we manually reviewed acoustic files from each day and selected four of the best weather days (see Appendix 1 for more details), resulting in a duration gradient of 1–4 recording days for each plot (Step 3, Figure 1).

Using gradients of these three parameters (intensity: every 3 min to every 60 min; day phase: dawn, morning, or both; and duration: 1–4 days), we created 60 subsetting scenarios from all possible combinations of gradients, and compared the bird richness and compositional dissimilarity yielded by each one (Figure 1). The advantage of looking at three dimensions simultaneously is to distinguish the difference between simply increasing time spent annotating (effort), and increasing the distribution of time (spread) over (a) one morning and (b) over multiple days. We generated a high number of subsetting scenarios to avoid prescribing one best‐performing scenario. Rather, the reader can observe how these factors interact and assess trade‐offs in accordance with their study aims and annotation budget.

We extracted 1‐min files of the most time‐intensive duration–day phase–intensity scenario, of which other scenarios could be subsampled. Using the GPS location of each plot center, we generated the exact sunrise time (defined as when the top edge of the sun appears on the horizon) for each plot day using the “suncalc” package (Thieurmel & Elmarhraoui, 2019) in R statistical computing environment (R Core Team, 2021). We then used the "seewave” and "tuneR” packages (Ligges et al., 2018; Sueur et al., 2008) to cut the long‐duration audio into 1‐min .wav files at 3‐min intervals, according to its relation to sunrise. This resulted in 160 files per plot, systematically sampled (20 one‐minute files × two dawn periods × 4 days). The gradient of files per duration–day phase–intensity combination was thus 1–160 files per plot, depending on the 60 scenarios (Figure 1). Nine plots had incomplete records due to water infiltration; these recorders were dried before functioning properly the following week in another plot and these plots were removed from subsequent analyses, resulting in 2720 files for manual annotation (17 plots × 160 one‐minute files).

### 2.4 Manual annotations

One observer (S.S.) identified all detectable bird species in each 1‐min file. Identifications were made by listening to audio as well as viewing spectrograms in Kaleidoscope Lite software v5.4.2 (Wildlife Acoustics, Inc., Maynard, MA, USA). Songs, calls, and, in certain cases, drumming were used to identify birds to species level whenever possible. Problematic identifications were checked by a second expert observer (J.P.). In the case of the *Certhia* and *Regulus* genera, it was not always possible to identify the species level, in which case only the genus was recorded (e.g., “*Certhia* sp.”), which were excluded from further analyses. The resulting dataset was a species list of all birds detected in each file, containing its associated plot, day, day phase, and time in relation to dawn.

### 2.5 Point count surveys

Point counts were also conducted in each study plot within 2 weeks before or after that plot's acoustic recording period. Point counts took place between half an hour after sunrise to 12:00 p.m. CET (always in the morning period). After arriving at the plot center, a 5‐min settle‐down period was employed (Gibbons et al., 1996). Each point count lasted 20 min, during which all birds detected aurally or visually were recorded at 5‐min intervals in order to reach an adequate sampling coverage to calculate detectability (Balestrieri et al., 2017; Basile, Asbeck, et al., 2021; Basile, Storch, et al., 2021; Sorace et al., 2000). Data were used from all detections without distance limit in order to be comparable to the detection radius of an acoustic recorder (Shaw, Müller, & Scherer‐Lorenzen, 2021). The resulting variable was species richness per plot observed during the 20 min. While the main objective of this study is to investigate the subset variables of duration, intensity, and day phase, we chose to include the point count data as a reference, given that point count surveys are still the standard traditional method for monitoring breeding birds in forest habitats (Green et al., 2010; Ralph et al., 1995), and will continue to be used in many study areas. A 20‐min point count is not directly comparable to subsampling from continuous audio recordings; point counts must take place over a continuous timeframe, and although PAMs have the advantage of capturing non‐consecutive moments, they lack the visual advantage of the point count method. Thus, we are simply comparing these methods in terms of effort–time spent, and the resulting richness/composition matrices each method, with all its advantages and disadvantages, produces.

### 2.6 Analysis

All analyses were made in R statistical computing environment. We investigated the effect of 60 recording scenarios on bird richness at two scales, the local plot level and across the entire study area. The local scale is defined as the 1 ha of forest around the plot center, a typical stand‐scale measurement in silviculture (citation). The scale of the entire study area is roughly 40 km^2^. Local‐scale data allowed us to create alpha diversity metrics that provide insights into what bird richness values any single recorder may yield in a given recording scenario. Data at the study area scale enabled the computation of beta diversity indices between sites, which provide comparisons of how different scenarios yield different total species richness within the study area.

For the local scale, we directly calculated the number of observed bird species at each site for each recording scenario (alpha diversity). We then computed the mean and standard deviation (*SD*), providing a local mean species richness per scenario. This metric is on a comparable spatial scale to the mean number of birds observed via the point count method.

To estimate the effect of different recording scenarios on bird richness at the study area scale, we created a community matrix for each plot and created sample‐size‐based rarefaction and extrapolation sampling curves for each one (Chao et al., 2014). We sampled community matrices (scenarios as rows and species as columns) with the “iNEXT” package (Hsieh et al., 2016), using incidence frequencies data to estimate Hill numbers of species richness (*q* = 0). The resulting variable was the cumulative number of unique species detected per recording scenario (observed species richness), 17 sites pooled. This analysis also provides a rarefaction curve for each scenario, analogous to a gamma diversity scale metric, or how many unique species can be detected within the entire study area as a function of increasing time spent on manual annotations, per recording scenario. The observed richness from rarefaction sampling (all plots pooled) was compared across scenario variables using a Student's two‐sample *t*‐test. Lastly, given that our plots are not uniformly distributed throughout the study area, we assessed our data structure for the influence of any potential spatial autocorrelation, and none was found (Appendix 1).

In order to compare species compositions between recording scenarios, we created a presence/absence community matrix from each scenario, using plots as rows and species as columns. We then made pairwise comparisons of the scenarios using beta diversity metrics in the “betapart” package (Baselga et al., 2022). Using the “beta. Pair” function and the “sorensen” family, we computed turnover (replacement) and nestedness dissimilarity indices from each incidence‐based pairwise comparison. The turnover metric is a dissimilarity index accounting for the replacement of species with different species, measured as Simpson pairwise dissimilarity. The nestedness index measures a separate feature of dissimilarity of two assemblages, how many species in one assemblage exist as part of another assemblage, and is measured as the nestedness fraction of Sorensen pairwise dissimilarity (Baselga et al., 2022). These dissimilarity indices allowed us to assess how much of the observed pairwise dissimilarities between two scenarios were (1) due mainly to differences in species richness yielded by each scenario (nestedness) because different scenarios inherently require differing amounts of manual annotation time; or (2) due simply to compositional dissimilarities between scenarios, despite any differences in species richness (turnover).

We also performed non‐metric multidimensional scaling (NMDS) analyses for certain scenarios in order to highlight certain differences reflected in the turnover values, using “family = euclidean" dissimilarity distance index. The “ggrepel” package was used to avoid overlapping centroids of species labels (Slowikowski, 2021). Plots were generated using the “ggplot2” package (Wickham, 2016).

## 3 RESULTS

### 3.1 Local bird richness

**FIGURE 2:**
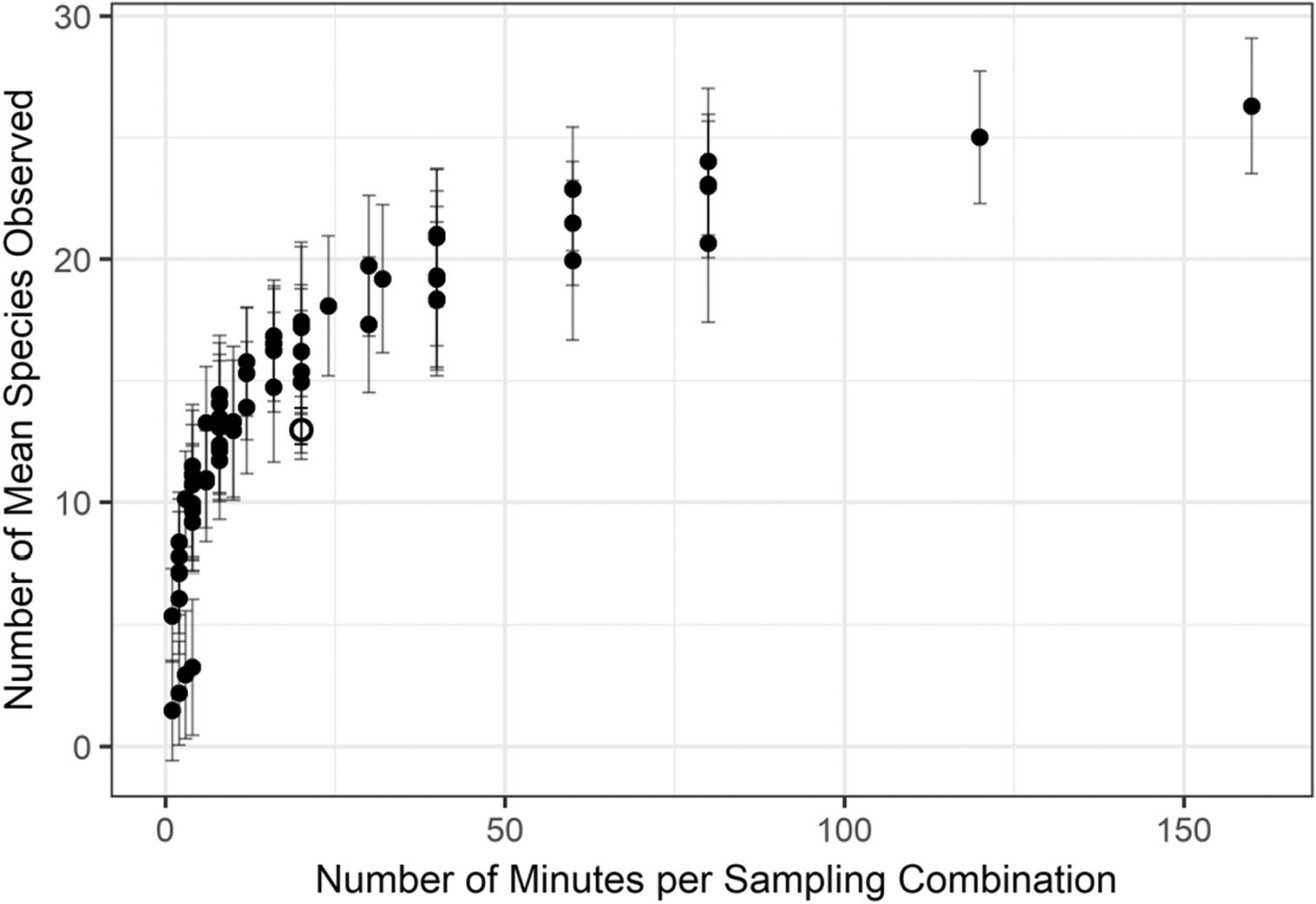
The number of mean species observed per annotation scenario. Open circle indicates the mean species richness observed from the point count method.

The mean species richness observed by each recording scenario ranged from 1.47 to 26.29 (Table 1). Mean number of birds identified increased asymptotically with the number of minutes required for bioacoustic ID per scenario (Figure 2). When directly comparing all scenarios requiring 20 min of effort, including point count surveys, all recording scenarios yielded higher mean species than the point count method, which produced a mean of 13.11 species (Table 1, black point in Figure 2). Thirty‐six scenarios observed higher bird richness than the point count method; 18 of these scenarios requiring ≤20 min of bioacoustic ID (bolded values in Table 1).

**TABLE 1:** Species richness at the local and study area scale across all annotation scenarios (intensity–day phase–duration combinations). No. minutes denotes how many 1‐min files (total manual annotation effort) correspond with each scenario. Scenarios are ranked by the number of species observed at both scales. Bolded values indicate observed richness values that exceed bird richness yielded by the point count method (bottom row).

Scenario				Local scale			Study area scale	
Intensity	Day phase	Duration	No. minutes	Observed mean	Observed *SD*	Rank observed	Observed	Rank observed
3	Dawn	1	20	**14.94**	**2.93**	**29**	**37**	**28**
3	Dawn	2	40	**18.35**	**3.16**	**16**	**43**	**12**
3	Dawn	3	60	**19.94**	**3.29**	**11**	**43**	**13**
3	Dawn	4	80	**20.65**	**3.26**	**10**	**43**	**14**
3	Morning	1	20	**15.35**	**3.59**	**27**	**36**	**30**
3	Morning	2	40	**19.29**	**2.87**	**13**	**44**	**10**
3	Morning	3	60	**21.47**	**2.55**	**7**	**46**	**7**
3	Morning	4	80	**23.06**	**2.61**	**4**	**46**	**8**
3	Both	1	40	**19.18**	**3.63**	**15**	**40**	**19**
3	Both	2	80	**23**	**2.96**	**5**	**50**	**1**
3	Both	3	120	**25**	**2.72**	**2**	**50**	**2**
3	Both	4	160	**26.29**	**2.78**	**1**	**50**	**3**
6	Dawn	1	10	12.94	2.88	39	31	40
6	Dawn	2	20	**16.18**	**2.58**	**25**	**37**	**26**
6	Dawn	3	30	**17.29**	**2.78**	**20**	**38**	**22**
6	Dawn	4	40	**18.29**	**2.85**	**17**	**38**	**23**
6	Morning	1	10	**13.29**	**3.1**	**35**	33	34
6	Morning	2	20	**17.41**	**3.08**	**19**	**41**	**16**
6	Morning	3	30	**19.71**	**2.89**	**12**	**43**	**11**
6	Morning	4	40	**21**	**2.67**	**8**	**44**	**9**
6	Both	1	20	**17.18**	**3.5**	**21**	**37**	**25**
6	Both	2	40	**20.88**	**2.85**	**9**	**47**	**4**
6	Both	3	60	**22.88**	**2.55**	**6**	**47**	**5**
6	Both	4	80	**24**	**3.02**	**3**	**47**	**6**
15	Dawn	1	4	9.94	2.36	49	26	50
15	Dawn	2	8	12.35	2.26	40	31	41
15	Dawn	3	12	**13.88**	**2.71**	**33**	32	36
15	Dawn	4	16	**14.71**	**3.08**	**30**	33	33
15	Morning	1	4	9.76	2.63	50	27	48
15	Morning	2	8	13.06	2.75	38	32	38
15	Morning	3	12	**15.29**	**2.73**	**28**	**39**	**20**
15	Morning	4	16	**16.53**	**2.37**	**23**	**40**	**18**
15	Both	1	8	**13.47**	**3.08**	**34**	32	37
15	Both	2	16	**16.24**	**2.54**	**24**	**38**	**21**
15	Both	3	24	**18.06**	**2.88**	**18**	**40**	**17**
15	Both	4	32	**19.18**	**3.05**	**14**	**42**	**15**
30	Dawn	1	2	7.12	2.47	55	24	54
30	Dawn	2	4	9.65	1.9	51	26	51
30	Dawn	3	6	10.82	2.46	46	26	49
30	Dawn	4	8	11.71	2.42	42	28	46
30	Morning	1	2	7.76	2.36	54	22	55
30	Morning	2	4	11.47	2.53	43	28	45
30	Morning	3	6	**13.24**	**2.33**	**36**	**36**	**31**
30	Morning	4	8	**14.41**	**2.43**	**31**	**37**	**27**
30	Both	1	4	10.71	3.06	47	27	47
30	Both	2	8	**14.06**	**2.01**	**32**	30	44
30	Both	3	12	**15.76**	**2.22**	**26**	**36**	**29**
30	Both	4	16	**16.82**	**2.3**	**22**	**37**	**24**
60	Dawn	1	1	1.47	2.07	61	11	61
60	Dawn	2	2	2.18	2.13	60	13	60
60	Dawn	3	3	2.94	2.63	59	15	59
60	Dawn	4	4	3.24	2.8	58	16	58
60	Morning	1	1	5.35	1.9	57	19	57
60	Morning	2	2	8.35	2.06	53	24	53
60	Morning	3	3	10.12	1.96	48	31	43
60	Morning	4	4	11.12	2.06	44	31	42
60	Both	1	2	6.06	2.28	56	21	56
60	Both	2	4	9.18	2.01	52	26	52
60	Both	3	6	10.94	2.01	45	32	39
60	Both	4	8	12.12	2.09	41	33	35
Point count			20	13.11	3.1	37	34	32

### 3.2 Bird richness across the entire study area

**FIGURE 3:**
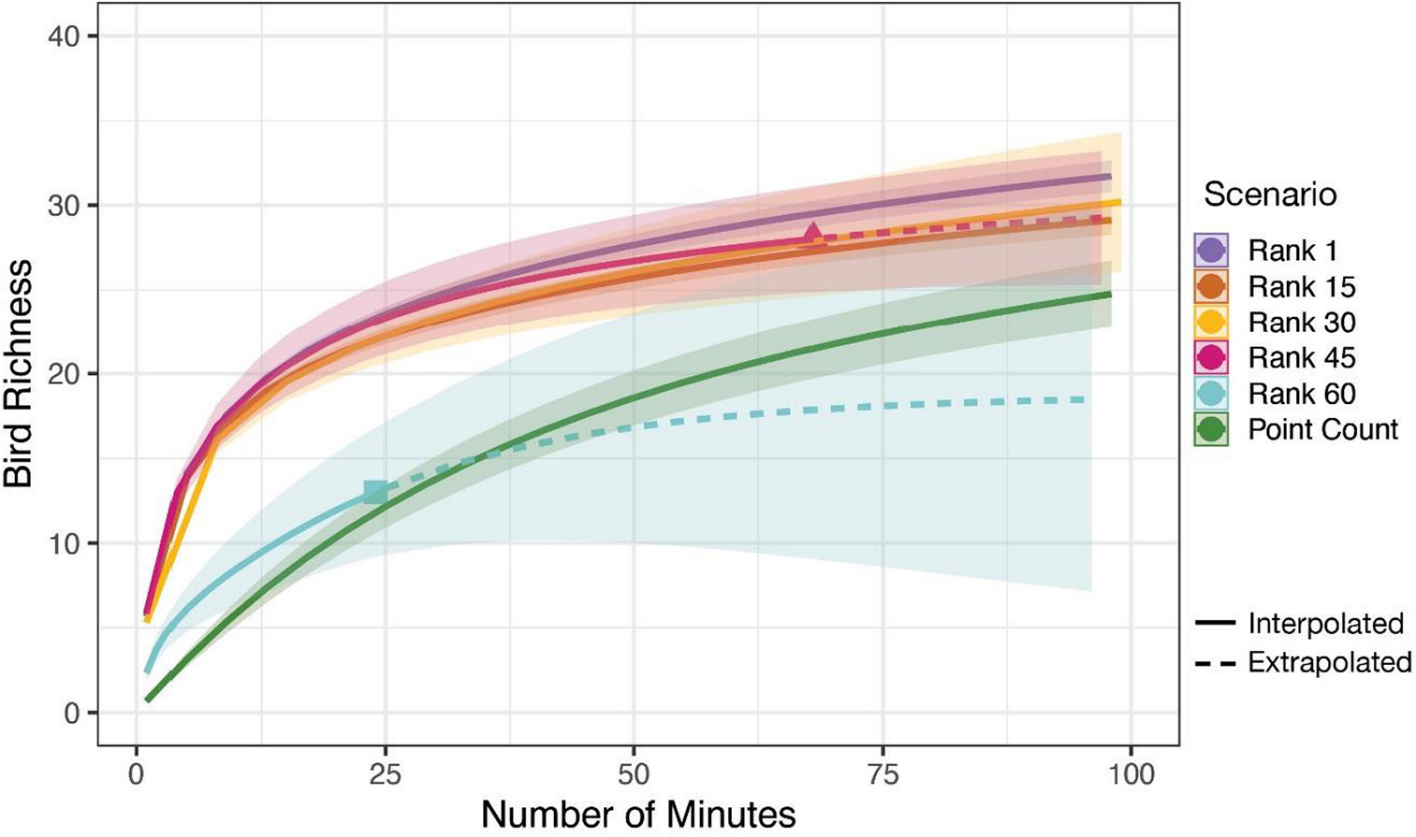
Species accumulation curves for four intensity–day phase–duration scenarios and the point count survey method. The four scenarios were selected by their quartile rankings of 1, 15, 30, 45, and 60 in the number of species observed through rarefaction sampling. Each curve represents the number of species detected from a given recording scenario (±95% CI) pooled from all 17 plots.

Observed species richness at the study area scale for each recording scenario ranged from 11 to 50 unique species (Table 1). The scenario yielding the highest species richness was with recordings at an intensity of every 3 min, across both day phases and for a duration of 2 days (50 species in 80 min). Manual annotations from 29 scenarios (bolded in Table 1) yielded greater bird richness than the traditional point count method, which yielded 34 observed species. Scenarios across three quantiles of observed species (ranked 1–45 of 60) accumulated new species at similar rates and greater than the rate of accumulation from the point count method (Figure 3). However, these scenarios required different amounts of manual annotation time. Of the five scenarios not requiring more than 20 min, all yielded higher species richness than the point count method.

**FIGURE 4:**
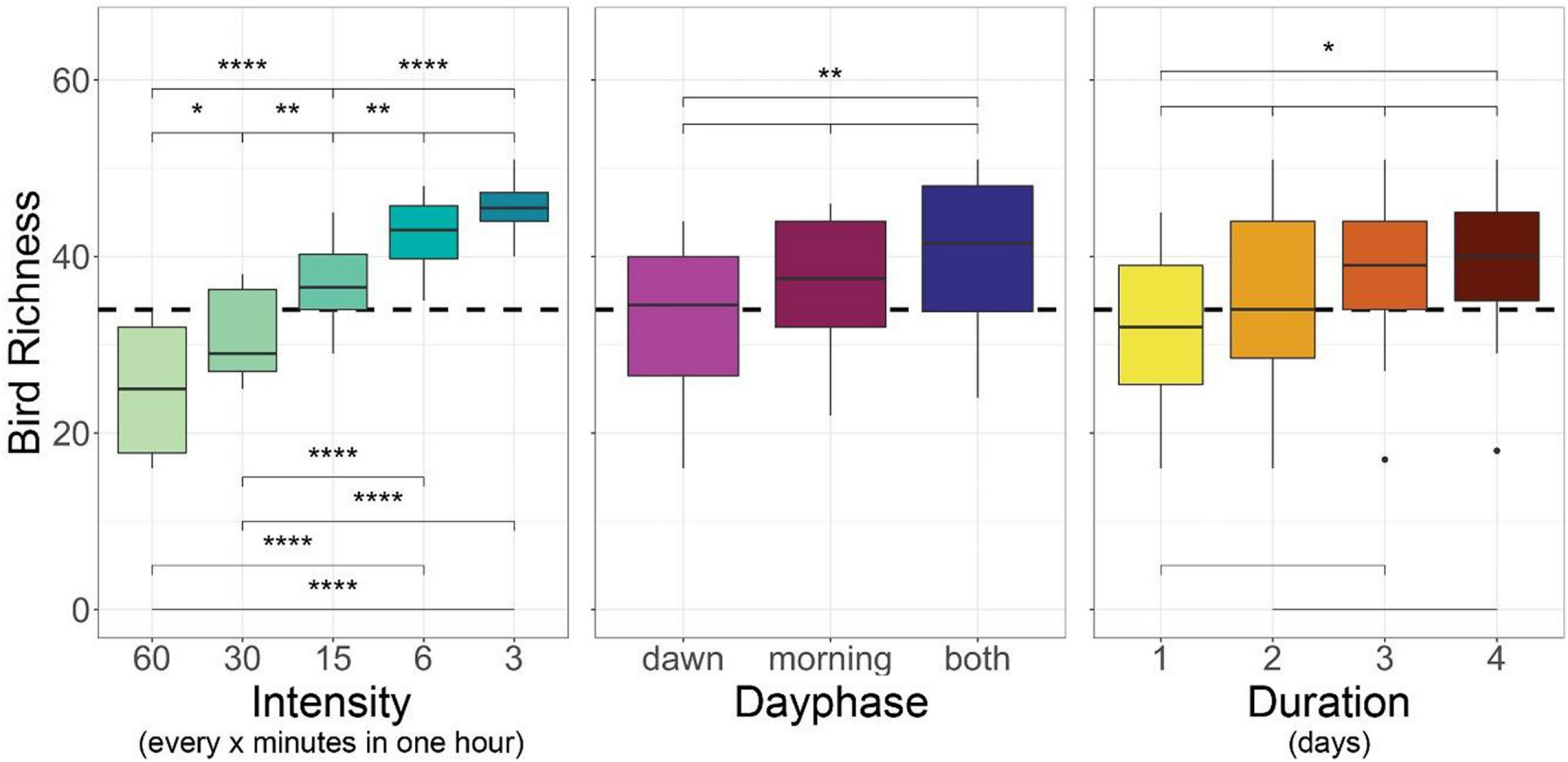
Boxplots of observed bird richness from 60 sampling scenarios, all plots pooled. Each panel depicts the same 60 scenarios, stratified by a different gradient (intensity, day phase, or duration). The dashed line represents the mean species richness observed by point counts across the same plots. Statistical significance at the 95% level is denoted by: * <.05, ** <.01, *** <.001, **** <.0001, and unmarked = not significant.

Across scenarios, species richness significantly increased with intensity (Figure 4). Richness was significantly higher when including both day phases compared to dawn alone, and was slightly higher if identifications were made in the morning, rather than dawn, period (Figure 4). Bird richness significantly increased with each added duration day up to 3 days; there was no difference in bird richness between 3 and 4 duration days.

### 3.3 Local assemblage composition

**FIGURE 5:**
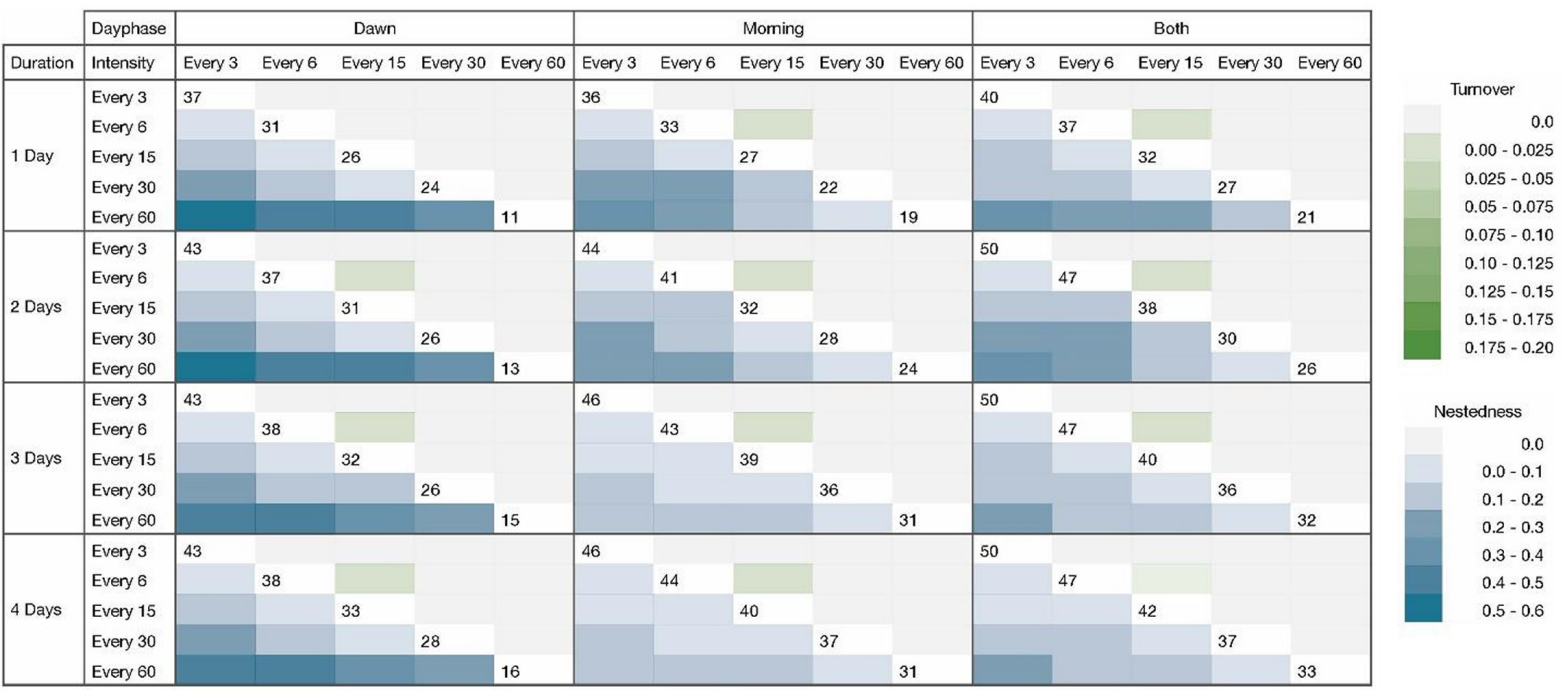
Pairwise comparisons of intensities (every 3, 6, 15, 30, or 60 min in 1 h) by all possible dawn phase–duration scenarios. Green and blue color ramps indicate increasing beta dissimilarity according to Sorensen turnover and nestedness values, respectively. Numbers at the intersecting white space of the same duration represent the species richness yielded by that scenario to aid in the comparison of richness‐related differences in nestedness versus turnover. Figures including beta diversity values in addition to color ramps in Appendix A.5.

Pairwise comparisons of intensities within all possible day phase–duration scenarios (Figure 5) show dissimilarities in assemblage composition among every 3‐, 6‐, 15‐, 30‐, and 60‐min recording intensities. The difference is mainly due to nestedness, as the blue values increase with higher subsetting intensity. According to the turnover values, there is little dissimilarity across intensity scenarios (values are mostly 0) that is not purely due to differences in richness (nestedness). At dawn, intensities of every 60 min were maximally dissimilar to the other intensities. This indicates that the number of species lost when comparing intensities depends on the day phase as well; every 60 min, at dawn only, yields the lowest species richness. Intensity is the most important variable in determining differences in species richness, expressed by the highest nestedness values (Figure 5) and higher significance between intensities (Figure 4).

**FIGURE 6:**
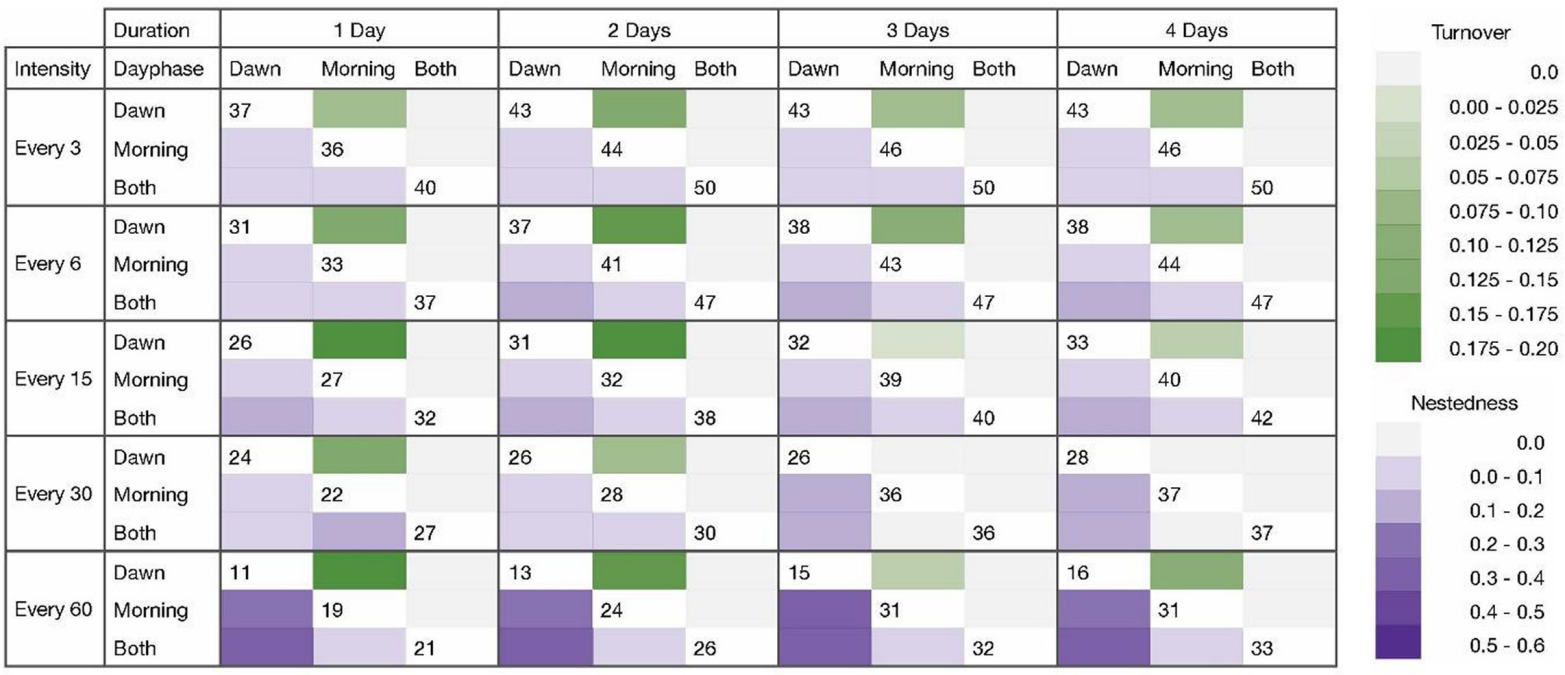
Pairwise comparisons of day phases (dawn, morning, or both) by all possible intensity–duration scenarios. Green and purple color ramps indicate increasing beta dissimilarity according to Sorensen turnover and nestedness values, respectively. Numbers at the intersecting white space of the same duration represent the species richness yielded by that scenario to aid in the comparison of richness‐related differences in nestedness versus turnover. Figures including beta diversity values in addition to color ramps in Appendix A.5.

**FIGURE 7:**
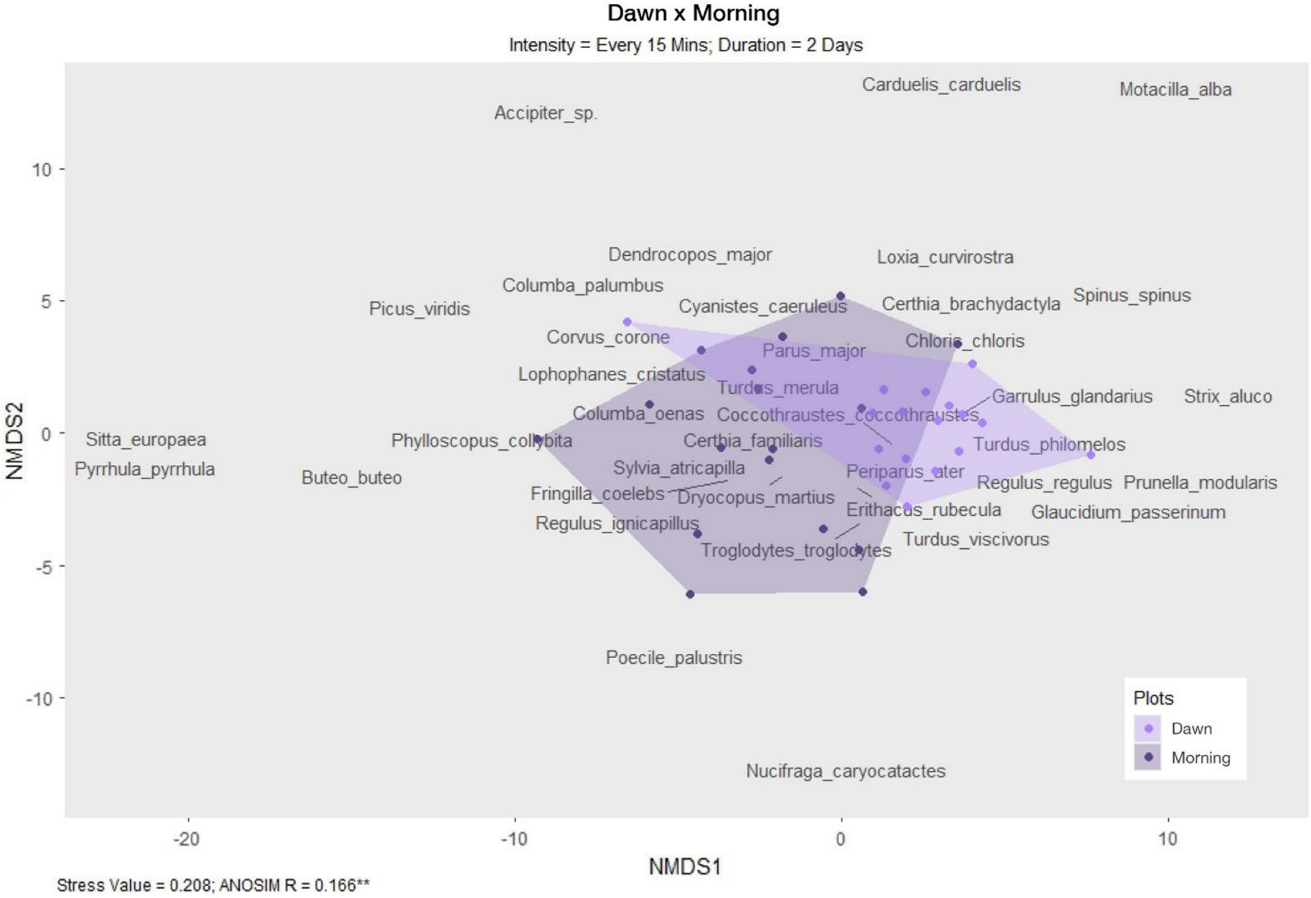
Non‐metric multidimensional scaling (NMDS) of species composition between dawn and morning recording periods in an intensity–duration scenario that produced almost equal bird richness observations (dawn = 31 species; morning = 32 species). The species names depict the distribution of species in two‐dimensional ordination space according to their co‐occurrence, and the purple dots depict how closely the 17 research plots (during either dawn or morning) are associated with a given species; each polygon links the outermost plots for the day phase periods.

**FIGURE 8:**
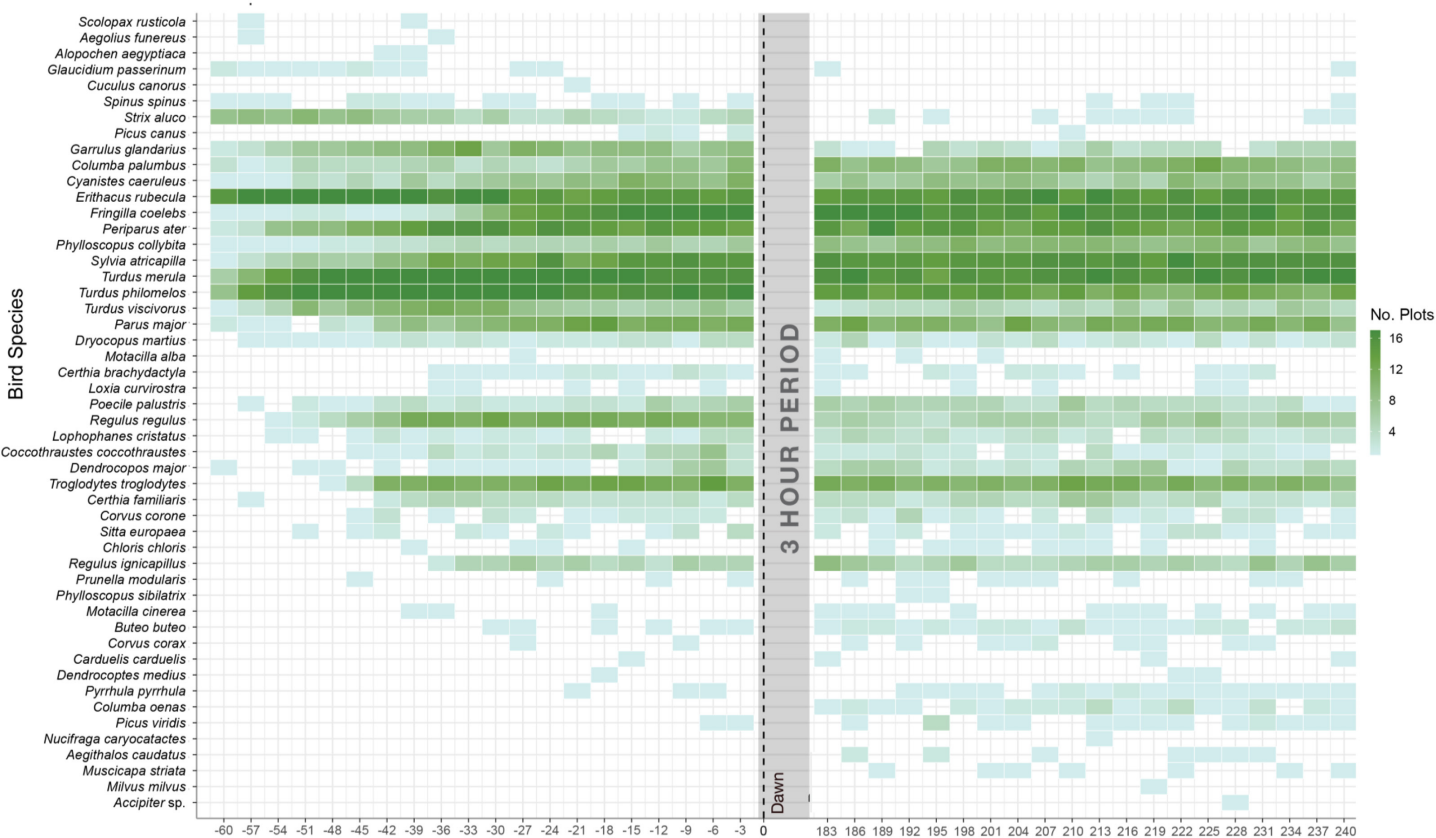
Bird occurrence relative to time since dawn taken from the highest sampling scenario (every 3 min intensity, both dawn and morning day phases, for a duration of 4 days). The color ramp displays the number of plots in which a given species was observed for a specific time since dawn, at 3‐min intervals (i.e., the darker the color, the more plots that species was observed at that particular time). If a species was observed in a given plot at a given time on more than 1 day, it was only counted once.

Pairwise comparisons of day phases across all possible intensity–duration scenarios (Figure 6) reveal both nestedness and turnover. Mild nestedness was found between dawn and morning sampling periods, indicating that there is an overlap between species detected at dawn versus morning, with the morning day phase yielding slightly more species. However, high turnover was found between scenarios sampled at dawn compared to the morning, suggesting that despite some similarity in species detected, distinctly different assemblage compositions were yielded from dawn and morning day phases. This effect is found across all durations and intensities, although it is stronger (darker green) in the durations of 1 and 2 days, and at high intensities of every 3 and 6 min. Moreover, in a scenario where species richness is almost numerically equal between dawn and morning (31 and 32 species, at every 15 min intensity and 2 days duration), assemblages were significantly different. Figures 7 and 8 highlight this observation that the morning period captures more species, whereas dawn recordings detect species unique to the dawn period. Day phase is the second‐most important variable in determining differences in species richness (nestedness), but the most important variable in determining differences in composition (turnover).

**FIGURE 9:**
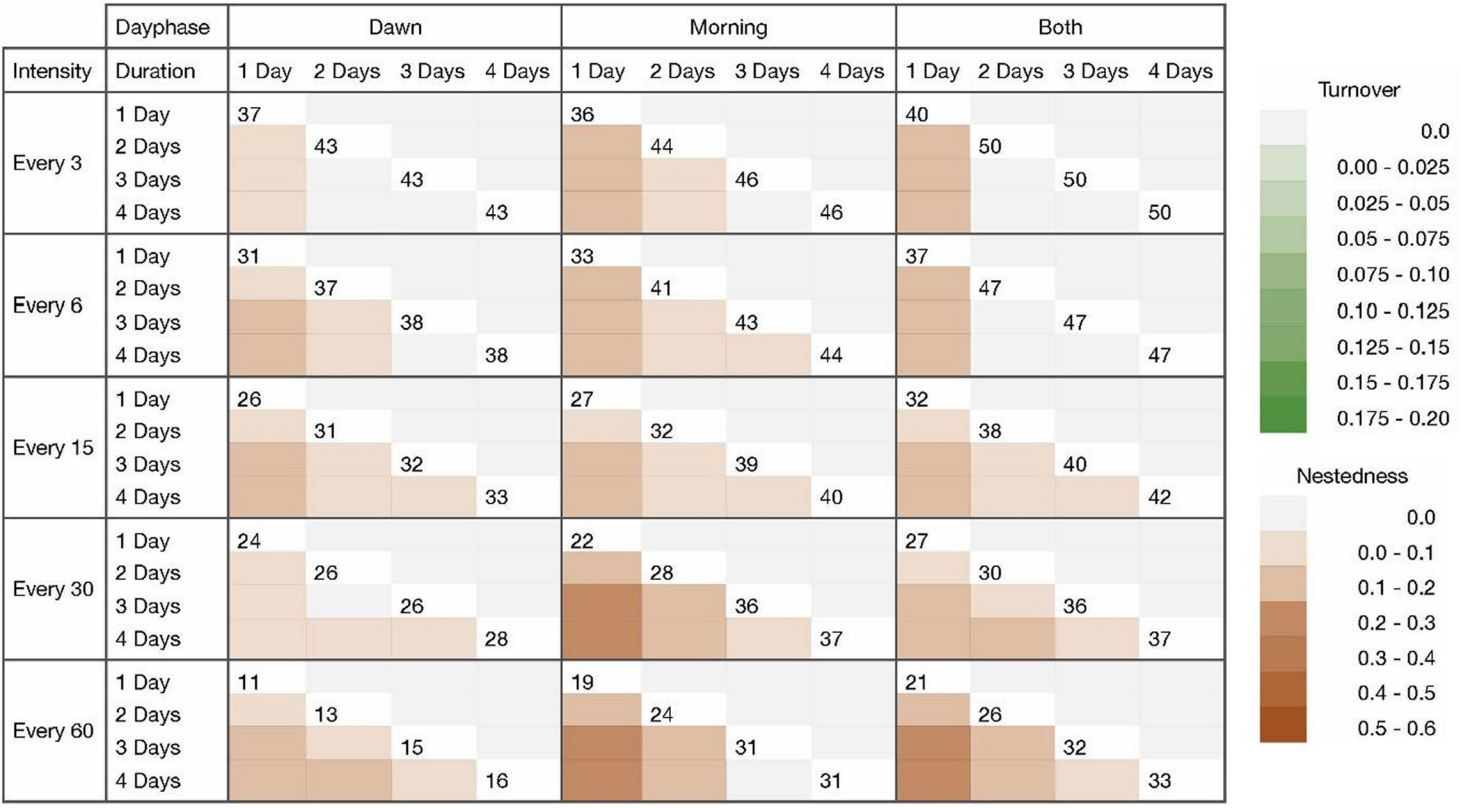
Pair‐wise comparisons of durations (1, 2, 3, or 4 days) by all possible intensity–duration scenarios. Green and orange color ramps indicate increasing beta dissimilarity according to Sorensen turnover and nestedness values, respectively. Numbers at the intersecting white space of the same duration represent the species richness yielded by that scenario to aid in the comparison of richness‐related differences in nestedness versus turnover. Figures including beta diversity values in addition to color ramps in Appendix A.5.

Comparisons of durations across intensity–day phase scenarios (Figure 9) show dissimilarities due to nestedness across almost all scenarios (orange cells), and no dissimilarities due to turnover (gray cells). The highest nestedness dissimilarity values are found between durations of 1 day versus multiple days, and this effect is strongest in the morning and dawn day phases and at weak intensities of every 30 and 60 min (darker orange cells). Thus, the duration variable interacts with both day phase and intensity variables: richness is higher in the morning day phase (and necessarily in “both” condition as well) and richness decreases at weak sampling intensities. There is no dissimilarity due to species turnover between duration scenarios, suggesting that the only duration‐related differences in the assemblage are due to an increase in the number of species detected (nestedness); different species are not detected on different days, but rather the unique number of species accumulated increases with increased investment of days included for manual annotation. Duration is the least important of the three variables in this study in terms of richness, as evidenced by the relatively lowest nestedness values, and the least important variable regarding species composition, as all turnover values were 0.

## 4 DISCUSSION

### 4.1 Bird richness

Whether manual annotation is used simply to directly obtain species richness estimates or to maximize novel vocalizations included in a training or validation dataset for machine learning classifications, we observed differences across all three variables in this study (intensity, day phase, and duration). The effect sizes, however, differed between variables, whereby increasing the intensity of subsets within a day had the strongest effect. Subsetting across both day phases maximized species accumulated, but not significantly so, as most species are adequately captured in the morning period alone. Increasing duration, or additional days of recording, had the smallest effect. Most species could be observed in the first 2 days of recording, in the morning period, and at a high recording intensity of every 3 or 6 min. Our results indicate that maximizing number of detected species would require subsetting at a high intensity, across both dawn and morning and for at least 2 days. If resources are limited—as they are in cases when researchers cannot or elect not to use automated classifiers—it appears that it is more important to increase the intensity of recordings within 1 day, rather than reducing intensity and increasing duration. Further, while most species can be observed in the morning, certain nocturnal and crepuscular species can only be observed in the dawn period.

Our study was performed on data originating from temperate forests and recorded during the peak of the breeding season. Our results indicate that intense subsetting during just 1 day is able to capture the majority of species, and an addition of 1 more day captures the great majority, and sometimes the maximum number of species able to be observed in this study. Wimmer et al. (2013) found that duration was not very important because the majority of species are accumulated within the first day, which we also found. In a review of terrestrial acoustic monitoring studies, Sugai et al. (2020) recommend annotating from more than 1 day of recordings, and our results partially agree; however, according to our data, this effect plateaus after 2 days. Similarly, previous studies found that increasing investment in more recordings over fewer days produced more accurate results than distributing that time over multiple days (La & Nudds, 2016; Wood et al., 2021), which our results strongly support.

We would like to highlight that recording across days in the early versus late breeding season may still be advantageous to fully characterize the breeding bird assemblage due to phenological differences between resident and migratory species (Südbeck et al., 2005). Particular consideration should be taken when designing studies targeting species important to conservation such as woodpeckers, owls, and other birds of prey, which breed only once per season, by carefully choosing a time period within the breeding season with the highest target species vocal activity. We did not consider this as problematic for our study, since many bird species in our system have multiple clutches, and our recorders, therefore, captured a range of phases during the breeding cycle. Our study focused on the effect of consecutive recording days rather than spreading those days across the entirety of the breeding season. Further, our study was able to go beyond the scope of previous studies (Drake et al., 2021; Wood et al., 2021), which could not disentangle the effect of additional minutes across the same period (intensity) from the effect of additional minutes across a wider period of time (coverage of day phases or the number of duration days). Our results indicate that both will result in higher species richness estimates (Figure 4), although multiday coverage matters less than intensity when trying to obtain a snapshot, or the majority, of breeding bird richness, as was the main intention of this study.

### 4.2 Assemblage composition

Similar to bird richness, compositions differed by intensity, day phase, and duration (in decreasing order of effect size, respectively). Compositional differences between scenarios can primarily be explained by nestedness, created by differences in sampling effort. Increased sampling effort increases the likelihood of detecting additional species, which necessarily increases the discrepancy in species richness from scenarios with lower sampling effort. The likelihood of detecting a given species is subject to variables outside the scale of intensity and day phase, such as breeding cycle (Gil & Llusia, 2020) and breeding status (Upham‐Mills et al., 2020), meteorological conditions and seasonal phenology (Slagsvold, 1977), noise pollution (Gil et al., 2015), and presence of conspecifics and interspecifics (Amrhein & Erne, 2006; Dolan et al., 2007; Foote et al., 2011; Klump, 2019; Liu, 2004; Xia et al., 2014). Because we do not expect a significant variation on these factors at the limited temporal scale in which we collected our data, nor do we expect these factors to vary with our intensity and day phase gradients, we attribute this nestedness mainly to increased richness with increased sampling effort.

However, the likelihood of detecting species during the dawn or morning period can be predicted to a larger degree based on those species' ecological traits, implying turnover in addition to nestedness as the underlying driver of compositional differences across day phases. Our results show that the dawn period, regardless of subsetting duration or intensity, yielded different species assemblages than the morning period (Figure 6), even when comparing scenarios with similar richness values (Figure 7). Generally, the morning period yields higher species richness, while dawn recordings disproportionately detect late‐calling nocturnal species, crepuscular species, and early callers (Figure 8).

It is well established that onset of vocalization varies per species (Allard, 1930; Allen, 1913). The common beginning of the sequence in European temperate forests is the robin (*Erithacus rubecula*), followed by the song thrush (*Turdus philomelos*), blackbird (*Turdus merula*), and the Eurasian wren (*Troglodytes troglodytes*) (Gil & Llusia, 2020). This predictable sequence is in part due to interspecific variations in light sensitivity per species, driven by eye size relative to body size (Thomas et al., 2002), feeding height (Berg et al., 2006), food guild (Chen et al., 2015), and differential responses to ambient light (Bruni et al., 2014; Dadwal & Bhatt, 2017; Miller, 2006). Our results aligned with these observations; in the earliest 1‐min acoustic files, mainly robins, song thrushes, and blackbirds were detected, sometimes together with infrequent nocturnal and crepuscular birds such as tawny owl (*Strix aluco*), Tengmalm's owl (*Aegolius funereus*), pygmy owl (*Glaucidium passerinum*), or Eurasian woodcock (*Scolopax rusticola*) (Figure 8). The robin and three thrush species (song thrush, blackbird, and mistle thrush [*Turdus viscivoru*s]) are all species sharing large eyes relative to their body size, and were also more frequently detected in the dawn than the morning period.

Other frequently detected species, such as the chaffinch (*Fringilla coelebs*) and coal tit (*Periparus ater*), began vocalizing later in the dawn period and their high vocal activity persisted throughout the morning period. This is perhaps due to high density and intra‐specific competition in these species, which according to the acoustic niche hypothesis (Krause, 1993) could favor the spread of their vocal activity across time. In the morning period, there were more frequent detections of multiple forest species which occur in low densities (e.g., northern raven [*Corvus corax*]), are highly specialized (e.g., middle‐spotted woodpecker [*Dendrocoptes medius*]), and threatened at the local level (wood warbler [*Phylloscopus sibilatrix*]; Bauer et al., 2016), or are resident birds that have a vocal activity peak earlier in the season, such as most woodpeckers (Billerman et al., 2020). Therefore, according to a researcher's aims—maximizing richness or the detection of particular species or groups—different phases of the morning are differently suited to those goals. Our results are generally most applicable to studies that do not target particular species, but rather aim to estimate the entire bird assemblage (at either the local or study area scale). Threatened species may require further testing and refining to adequately monitor population trends over time (e.g., Pérez‐Granados et al., 2018).

Nevertheless, interpretation of these results should be made with care for species that were detected only on one or few plots. Similar to bird composition, Wood et al. (2021) investigated assemblage structure, specifically the proportion of rare species in an assemblage, and the probability of different recording scenarios over‐ or underestimating the number of rare species in that assemblage. The greater the number of rare species occurring in a bird assemblage, the more species richness was underestimated across sites. Assemblages in our study area are comprised of few rare species and mainly of species with generalist habitat requirements and high vocalization rates, as is typical of managed Central and Western European forests (Mikusiński et al., 2018). There were rare observations in our dataset (very light‐blue squares in Figure 8), although that was in some cases due to non‐forest species being observed by chance (e.g., one observation of Egyptian Goose [*Alopochen aegyptiaca*]), which conveys no information about the rarity of a species using a forested habitat. However, relatively few observations of others species do indeed indicate their rarity, and results from this study can help target and increase the probability of their detection. For example, from manual annotations, we observed several regionally rare or more locally occurring forest species (Bauer et al., 2016) in several plots (Figure 8), such as the stock dove (*Columba oenas*), middle‐spotted woodpecker, spotted flycatcher (*Muscicapa striata*), and spotted nutcracker (*Nucifraga caryocatactes*). Our data are limited in the conclusions that we can draw about species rarity because without accurate abundance estimates, we cannot disentangle the variations in vocalization rates by species (Balantic & Donovan, 2019) and their abundance in our study region. Our results are directly applicable to other studies in central European forests with mostly generalist bird assemblages using habitats with a high proportion of conifers, active silvicultural management, and in landscapes with a long history of anthropogenic use. Strictly protected forests, old‐growth forests, or forests near water bodies would likely contain more specialist species with differing detection probabilities and/or vocal activity rates.

### 4.3 Manual annotation compared to the point count method

Comparing all annotation scenarios at the local and study area scale with equal to or less than the survey effort of point counts, all scenarios yielded higher richness values than point counts. However, this was not the primary question in our study, and this strong difference is mainly due to leveraging the capability of recorders to distribute audio samples over non‐consecutive time periods, while point counts necessitate consecutive minutes of survey effort. Other explanations are the passive quality of recorders, which negate flushing/avoidance effects created by human observers (Darras et al., 2018) and can generally be in the field at times when observers cannot, such as night or dawn. Other studies comparing point counts to identifications from an audio file found either similar outcomes between methods (Alquezar & Machado, 2015; Castro et al., 2019; Darras et al., 2018; McGuire et al., 2011; Van Wilgenburg et al., 2017; Yip et al., 2017) or that recorders outperformed humans (Borker et al., 2015; Digby et al., 2013; Haselmayer & Quinn, 2000; Hutto & Stutzman, 2009; Klingbeil & Willig, 2015; Sedláček et al., 2015; Shaw, Hedes, et al., 2021; Tegeler et al., 2012; Venier et al., 2012; Zwart et al., 2014). However, most of these studies compared consecutive recordings with consecutive point count minutes, where the visual advantage of point counts is maximized and the temporal distribution advantage of recorders is nullified. Additional advantages of manual annotations of acoustic data include the minimization of observer bias and the possibility for an observer to replay recordings, isolate particular frequency bands or time segments, and view recordings as spectrograms. However, additional effort should be accounted for when considering the use of these benefits (approximately 2:1 effort ratio; Wimmer et al., 2013). Drawbacks include technical failure, lack of visual confirmation of species and their associated microhabitats (Shonfield & Bayne, 2017), and identification uncertainty due to the occurrence of vocal mimicry in some bird species or truncated vocalizations. Given those considerations, the ability to estimate abundance (Bombaci & Pejchar, 2018; Van Wilgenburg et al., 2017) and create additional metrics such as multiple birds per file, occurrence frequency, and vocal activity rate are promising (Pérez‐Granados & Traba, 2021; Shaw, Müller, & Scherer‐Lorenzen, 2021).

## 5 CONCLUSION

Efficient allocation of annotation effort for species‐level identification is important for producing bioacoustic species richness estimates or training and validating more scalable automated classification algorithms. Based on our results, we recommend that researchers in central European forests whose primary goal is to maximize number of unique detected species prioritize recording intensity (number of minutes within an hour) in the morning period (in our case yielding 80% of known species from 20 min of audio). However, additional unique species can be added by including a dawn recording period on the same day (88% of species from 40 min), and maximum relative richness was obtained by adding 1 more day at the same intensity–day phase combination (100%); however, this doubles the time from 40 to 80 min per plot. Further, we urge researchers to also consider the species composition that their subsetting approach yields. If a survey's aim is to detect the most species possible, different design parameters will produce dissimilar assemblage compositions, potentially omitting rare or crepuscular species, species representing additional functional groups and natural history guilds, or species of higher conservation concern. We do not recommend one particular subsetting regime for all annotation objectives, but simply present multiple scenarios for researchers to understand how intensity, day phase, and duration interact in order to identify the best subsetting regime for one's particular research interests. Generally, however, we expect the intensity and day phase parameters to be important in any habitat with a distinct bird breeding season and a dawn chorus phenology, respectively. It is our hope that these data prove useful in optimizing breeding season acoustic survey programs for temperate forested regions and allow future studies to make efficient use of resources to achieve their conservation, monitoring, and research goals.

## AUTHOR CONTRIBUTIONS

**Taylor Shaw:** Conceptualization (lead); formal analysis (lead); investigation (equal); methodology (lead); visualization (lead); writing – original draft (lead); writing – review and editing (equal). **Sina‐Rebekka Schönamsgruber:** Data curation (lead); formal analysis (supporting); investigation (equal); visualization (supporting); writing – review and editing (supporting). **João M. Cordeiro Pereira:** Conceptualization (supporting); data curation (supporting); investigation (supporting); writing – review and editing (equal). **Grzegorz Mikusiński:** Conceptualization (supporting); supervision (supporting); writing – review and editing (equal).

## FUNDING INFORMATION

This study was part of the Research Training Group ConFoBi (GRK 2123/2), which is funded by the German Research Foundation (DFG). This work was also funded by State Graduate Funding of Baden‐Württemberg, through the University of Freiburg's International Graduate Academy (IGA).

## CONFLICT OF INTEREST

The authors declare no conflict of interest.

## Figures and Tables

**FIGURE A1:**
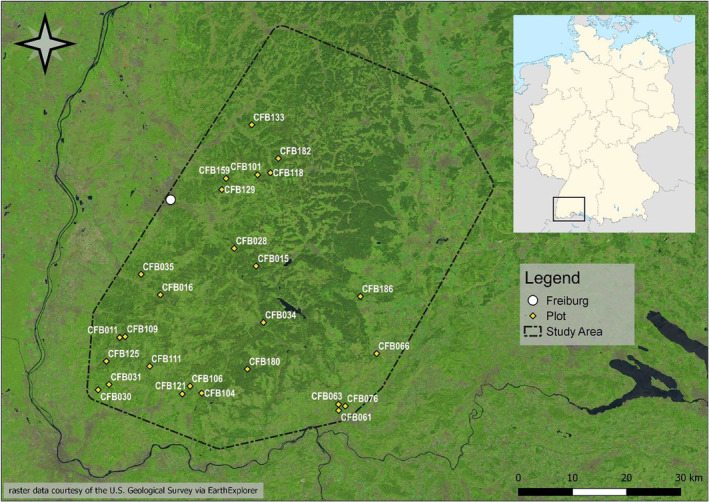
Original plot selection for the study conducted from April to June 2021 in the southern Black Forest, Germany. The study area of the original 135 plots is delineated by the black polygon shape, and the 26 plots that met inclusion criteria are labeled therein.

**FIGURE A2:**
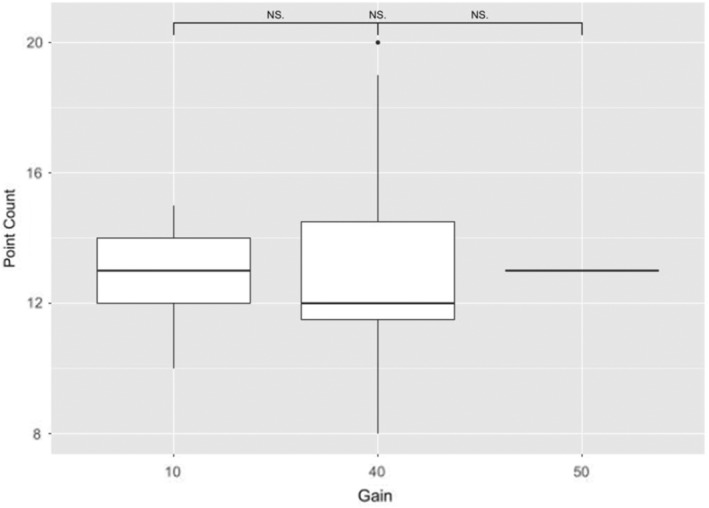
Boxplot depicting point count bird richness from the 17 plots in this study. Non‐significant tick marks are from a Wilcoxon rank sum test at the 95% significance level.

**FIGURE A3:**
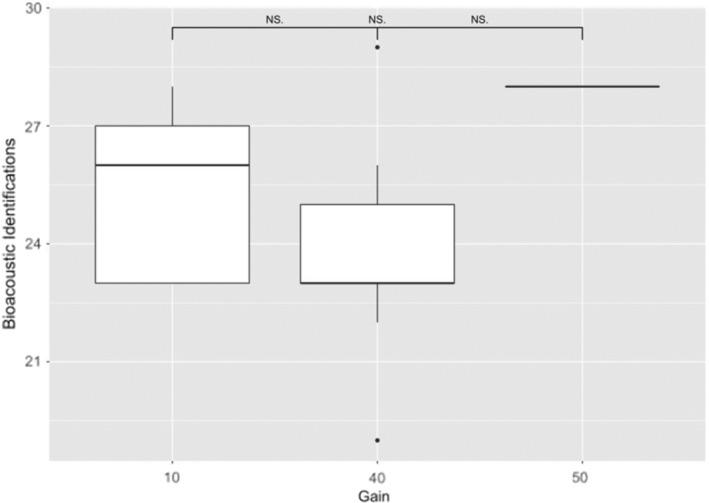
Boxplot depicting bioacoustic identification bird richness from the 17 plots in this study. Non‐significant tick marks are from a Wilcoxon rank sum test at the 95% significance level.

**FIGURE A4:**
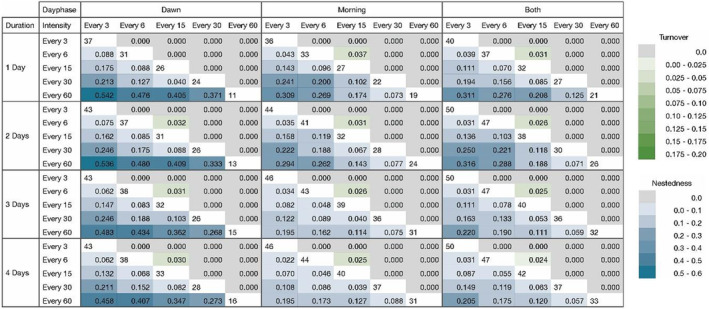
Pairwise comparisons of intensities (every 3, 6, 15, 30, or 60 min in 1 h) by all possible dawn phase–duration scenarios. Green and blue color ramps indicate increasing beta dissimilarity according to Sorensen turnover and nestedness values, respectively. Numbers at the intersecting white space of the same duration represent the species richness yielded by that scenario to aid in the comparison of richness‐related differences in nestedness versus turnover.

**FIGURE A5:**
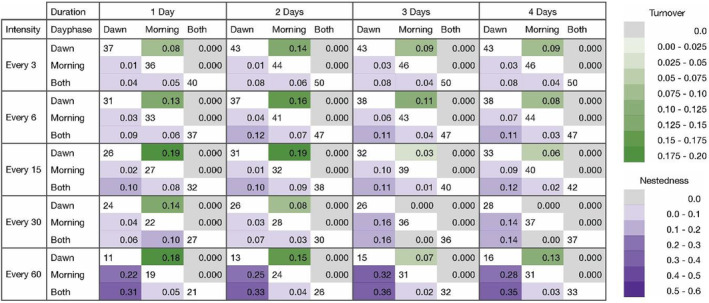
Pairwise comparisons of day phases (dawn, morning or both) by all possible intensity–duration scenarios. Green and purple color ramps indicate increasing beta dissimilarity according to Sorensen turnover and nestedness values, respectively. Numbers at the intersecting white space of the same duration represent the species richness yielded by that scenario to aid in the comparison of richness‐related differences in nestedness versus turnover.

**FIGURE A6:**
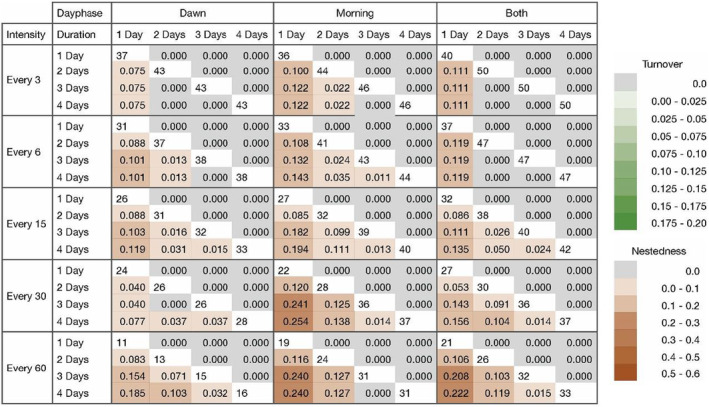
Pairwise comparisons of durations (1, 2, 3, or 4 days) by all possible intensity–duration scenarios. Green and orange color ramps indicate increasing beta dissimilarity according to Sorensen turnover and nestedness values, respectively. Numbers at the intersecting white space of the same duration represent the species richness yielded by that scenario to aid in the comparison of richness‐related differences in nestedness versus turnover.

## Data Availability

The data that support the findings of this study are openly available at Open Science Forum (OSF), https://osf.io/uq3cv/.

## APPENDIX 1

### Study area

The original 26 plots (Figure A1) were selected according to the following criteria, made available by a 2017 forest inventory (Storch et al., 2020):

- *Tree species richness gradient*. By this, we mean a sufficient mix of broadleaved and coniferous trees in a coniferous‐dominated forest based on basal area. All plots contained >5% beech and < 79% spruce.
- *Understory vegetation, dead wood, and age variation*. These are important structural components for different bird guilds (Balestrieri et al., 2015; Basile, Asbeck, et al., 2021; Basile, Storch, et al., 2021; Hinsley et al., 2009; Radu, 2006; Schall et al., 2018), and we did not want particular plots with extremely high or low values in one of these categories to confound the variables of primary interest. Plots met inclusion criteria if they contained >5% shrub layer cover (excluding “cathedral plots” with old beech trees and no understory), <7% basal area of standing dead wood (which excluded outlier plots with bark beetle and windthrow damage), and a gradient of structural complexity (variation in diameter at breast height from 77 to 246 mm).

### Accounting for variation in gain settings

In Table A1, the gain setting for each BAR is listed. Eleven of 17 devices were set to 40, five were set to 10, and one was set to 50 dB. We checked if our dependent variable, bioacoustic identifications, was affected by this error. Figure A2 depicts bird richness across the 17 plots in our study, derived from the point count method. According to this monitoring method, there was no difference between mean bird richness per plot among the plots that had recorders with different gain settings. Figure A3 depicts bird richness derived from bioacoustic identifications across the same plots. While there are differences in mean bird richness, they are not statistically significant. Further, they do not follow a pattern that would suggest the pattern is due to differences in gain. Lower gain settings reduce the recorder detection space, and if were to produce a significant effect, it would reduce the mean number of birds observed for recorders with lower gain settings. The four recorders with a gain of 10 should therefore have a lower mean value of bird richness. However, Figure A3 depicts higher bird richness for lower gain values, suggesting that the differences in non‐significant patterns are due to random variation in bird richness across those plots, which the bioacoustic method could detect more finely than the point count method.

**TABLE A1:** The list of BARs used in each study plot, the corresponding gain setting used, and resulting bird richness from both point counts and bioacoustic identifications per plot.

Plot	Gain	Point count richness	Bioacoustic identification richness
CFB030	10	15	28
CFB104	10	10	26
CFB125	10	13	27
CFB011	10	12	23
CFB034	10	14	23
CFB035	40	19	26
CFB061	40	15	24
CFB063	40	20	29
CFB076	40	12	22
CFB118	40	8	23
CFB159	40	14	23
CFB016	40	11	23
CFB106	40	9	23
CFB109	40	12	25
CFB182	40	12	19
CFB186	40	14	25
CFB121	50	13	28

### Selection “best weather days”

After removing the first and last day from each recording period, 6 recording days remained per plot, from which we chose the 4 best weather days. We define best weather days as the only 4 days without rain if there were 2 rainy days within the series. If there were more than 2 days of rain in a series, we chose to include the rainy days with the least amount of rain in the morning, which is the time period from which we would make bioacoustic identifications. We achieved this distinction by reviewing the files for the presence of only drips, and not active rainfall, indicating the rain event was over. We also looked for the presence of birdsong, which would indicate that the rain was not so intense that it would (1) mask bird vocalizations; or (2) be so intense that birds would not be vocalizing. In the event that all 6 days were “good weather days,” we selected the days furthest apart from one another to minimize temporal autocorrelation between days, thus maximizing the potential effect of duration, which this study investigates. For example, if a BAR was installed on a Monday and retrieved on the following Monday, Tuesday–Sunday would have been the 6 available days in this series. If there were no poor weather events detected, Tuesday, Thursday, Saturday, and Sunday were used. If more than one rain event was detected in a series, they typically happened consecutively, thus preserving a similar degree of temporal autocorrelation.

### Testing the spatial autocorrelation of plots

In order to test spatial autocorrelation between our plots, we created a generalized linear mixed model using the “glmmTMB” package (Brooks et al., 2017), including all data used in this study (60 scenarios × 17 plots, *n* = 1020). The dependent variable was species richness per plot per scenario, and the independent variables were the three variables of interest in our study (day phase and duration) as factors. The plot was used as a random factor to account for repeated measurements per plot with different scenarios. We next extracted residuals from the model, aggregated them by our random‐effect grouping term (plot), and performed the Moran's *I* distance‐based autocorrelation test on the aggregated residuals in relation to their *x*–*y* coordinates. We did so using the “testSpatialAutocorrelation” function in the “DHARMa” package (Hartig, 2022).

Our model did not show significant autocorrelation via the Moran's *I* test: observed = 0.040511, expected = −0.062500, *SD* = 0.123486, and *p*‐value = .4042. From this result, we can reject the alternative hypothesis that there is spatial autocorrelation in our data structure, and continued our analyses assuming spatial independence of our research plots.

Further, our plots are a minimum distance of 750 m from one another, greatly exceeding the recommended distance of 200 m between survey points for breeding birds to ensure the independence of results (Gregory et al., 2004).

The implications for our two spatial scales are that we consider the results at the local scale (means per scenario) to be comprised of 17 spatially independent replicates, from which we created alpha diversity metrics (bird species richness). Results at the study area scale (total unique species observed per scenario) simply aggregate all species found throughout the 17 plots, enabling beta diversity analyses between plots (pairwise dissimilarity indices of nestedness and turnover of each scenario's bird assemblage composition).
